# Comparative Study of Cooperative Platoon Merging Control Based on Reinforcement Learning

**DOI:** 10.3390/s23020990

**Published:** 2023-01-15

**Authors:** Ali Irshayyid, Jun Chen

**Affiliations:** Department of Electrical and Computer Engineering, Oakland University, Rochester, MI 48309, USA

**Keywords:** vehicle platoon, merging, deep reinforcement learning, proximal policy optimization, fuel consumption

## Abstract

The time that a vehicle merges in a lane reduction can significantly affect passengers’ safety, comfort, and energy consumption, which can, in turn, affect the global adoption of autonomous electric vehicles. In this regard, this paper analyzes how connected and automated vehicles should cooperatively drive to reduce energy consumption and improve traffic flow. Specifically, a model-free deep reinforcement learning approach is used to find the optimal driving behavior in the scenario in which two platoons are merging into one. Several metrics are analyzed, including the time of the merge, energy consumption, and jerk, etc. Numerical simulation results show that the proposed framework can reduce the energy consumed by up to 76.7%, and the average jerk can be decreased by up to 50%, all by only changing the cooperative merge behavior. The present findings are essential since reducing the jerk can decrease the longitudinal acceleration oscillations, enhance comfort and drivability, and improve the general acceptance of autonomous vehicle platooning as a new technology.

## 1. Introduction

Most highways in urban areas are congested to some extent, reducing mobility and increasing travel times for drivers, which results in wasted fuel consumption and additional traffic emissions. Vehicle platooning is a promising road management system to reduce congestion, fuel consumption, and accidents [1,2]. By platooning, multiple partially or fully automated vehicles are arranged in a train-like formation, with the lead vehicle at the front and multiple following vehicles. Vehicles in a platoon are coordinated to move at the same speed while maintaining a desired inter-vehicle distance [3]. In some road situations, the platoon has to perform lateral transitional maneuvers essential for safety and driving efficiency, such as joining, merging, and leaving the platoon [4].

The way vehicles merge significantly impacts road safety and traffic conditions. For human drivers, it is known that the zipper merge is one of the best approaches to go about when one of the lanes is closed. According to [5], the zipper merge strategy can reduce the overall length of traffic backup by up to 40%. Though counterintuitive, vehicles on the terminating lane should not ask to merge too early but rather wait until its lane is about to end to take turns merging to the open lane. Such strategy can ensure that all of the road capacity is fully utilized. However, platoon merging for connected and automated vehicles (CAV) is still challenging due to different interpretations of standards, and wireless communication [6,7]. In addition, the platoon merging involves multi-CAV interactions, requiring accurate real-time control with limited inter-vehicular communication cost [8]. Furthermore, according to [9], the merging approach for cooperative driving is still challenging to complete because of the significant interference created by unintentional vehicles interacting with the platoon resulting in aborting the platoon merge maneuver.

To address these challenges, Reference [10] investigated the cooperative merging algorithms in the presence of human-driven vehicles using a model predictive control (MPC) scheme. The controller solves the sub-problem of triplets of vehicles and finds smooth motion trajectories for different types of triplets. Reference [11] investigated the cut-in situations when a human-driven vehicle changes lanes into a platoon of CAVs. During the study, thirty-seven drivers participated in driving the human-driven vehicle using a driving simulator. The suggested inter-vehicular gap is 15 m to prevent human drivers from trying to cut in, which will create congestion at the merging point. Conversely, 30 m is found to be comfortable and easier to merge into the platoon. Authors of [12] produced an optimal speed profile during platoon merging on highway on-ramp to reduce the energy consumption of battery electric vehicles (BEVs), while [13] evaluated three different interactions protocols of two platoons facing lane-merging to optimize the total time to complete the maneuver, the string platoon length, and the average speed of the platoon. Two platoons of five vehicles were used in the simulation environment SUMO. To summarize, the motivation and benefits of this paper are as follows.

- Implementing platoons can decrease traffic congestion, reducing the time spent traveling, the amount of fuel consumed, and traffic emissions.
- The position where the platoon starts to merge in a road reduction significantly affects the vehicle’s performance.
- Improve road capacity utilization by decreasing the inter-vehicular gaps without raising safety concerns.
- Platoons can reduce traffic oscillations by eliminating extreme acceleration and deceleration.
- Studying the fuel consumption of electric vehicles will improve the driving range of such technology, which will help its adoption.

In this paper, the optimal merging technique is investigated. Specifically, the aim of this paper is to find the best distance between the merging vehicle and the start of the merge at which each vehicle in a platoon initiates a merging request in order to achieve the best possible performance. Several objectives are considered, including reducing travel time, ensuring passengers’ comfort by eliminating extreme acceleration and deceleration, improving environmental friendliness by decreasing energy consumption, etc. A model-free reinforcement learning (RL) approach, i.e., proximal policy optimization (PPO) [14,15], was used to learn the optimal merging policy. PPO is a deep reinforcement learning (DRL) method that falls into policy gradient methods, which is based on actor–critic methods. The actor maps observation to actions, and the critic returns the rewards estimate of the observation received. It starts by collecting the trajectories using the current stochastic policy. Then the cumulative rewards and the advantage estimates are calculated based on the interactions collected and used to update the actor and critic neural networks. Finally, using the clipping function introduced in PPO, the policy is updated for multiple epochs without the concern of changing the policy too far from the current policy.

The contribution of this paper is as follows.

- First, a DRL-based learning framework is proposed to learn the optimal merging behavior for fully connected and autonomous platoons.
- Second, a simulation environment is constructed to facilitate the DRL training. Low level longitudinal and lateral controllers are implemented based on PID, with the Beizer curve being used for path planning for the lane change.
- Thirdly, several metrics are studied to quantify the performance of different merging strategies, including time to merge, jerk, maximum jerk, speed, and energy consumed.

The remainder of this paper is organized as follows. Section 2 reviews relevant literature and highlights the contribution of the proposed work. The vehicle model and platoon’s configuration are presented in Section 3, while a preliminary study on RL is given in Section 4. The proposed DRL-based merging framework is discussed in Section 5, and numerical results are discussed in Section 6. Section 7 concludes the paper.

## 2. Literature Review

Several methods have been previously suggested to solve the CAV joining, merging, and leaving the platoon. Table 1 summarizes recent articles regarding platoon control maneuvers. In [16], a platoon merging approach was proposed using distributed MPC to reduce fuel consumption. Reference [17] studied the effect of lane change maneuvers on vehicle platooning. Evaluation of the lateral trajectories of platoons when one/several vehicles merge from the adjacent lane into the main vehicle platoon was discussed in [18]. The merging of heterogeneous vehicular platoons was studied in [19], where the authors concluded that the proposed controllers’ performance is satisfactory, but a more complicated scenario is needed for testing. A distributed MPC was proposed to generate the merging trajectories, while a linear quadratic regulator (LQR) controller was designed to create a gap for the merging platoon. Reference [20] proposed a novel PID controller for heavy-duty vehicle platoon maneuvers, while the authors of [21] showed that using cooperative adaptive cruise control (CACC) for highway-merging scenarios improves traffic-flow stability and efficiency. However, the proposed approach of [21] only considered longitudinal vehicle control, which means only vehicle speed would be automated using vehicle-to-vehicle communication, while the active steering of the ego vehicle and the behavior of the surrounding vehicles were not considered.

The most relevant study to this paper is [22], where the authors proposed a distributed controller utilizing a state feedback law to guarantee a collision-free vehicle merging when facing a road reduction. However, the lateral movement of the merging vehicles was not included in the analysis. Furthermore, the optimal merging location (as measured by the distance between the end of the lane and the merging vehicle) at which the merging vehicle should initiate a merge request was not investigated but assumed. Note the merging location can significantly affect the system level efficiency and safety, and the optimal merging location is not obvious given a particular scenario. Therefore, the assumption that the optimal merging location is known, as made by [22], is not realistic. This paper fills this gap by utilizing RL to interactively learn the optimal merging location to improve fuel efficiency and ride comfort.

The learning capacity of RL has recently been significantly improved by utilizing deep neural networks (DNNs), and the resulting deep reinforcement learning (DRL) algorithms have shown a human-level performance on complicated tasks such as playing Go, Chess, and Shogi [23,24]. With the increasing accessibility of low-cost, high-performance computing technology, DRL has been effectively applied to various areas. With the help of neural networks as function approximators, DRL can handle large dimensions of state or action space [25,26,27,28,29,30], which is the case with autonomous vehicle platoons [31]. Using a model-free DRL algorithm eliminates the need to model the environment’s complicated dynamics (the transition function/probability distribution). Instead, agents can learn from interacting with the environment for millions of time steps, and the more complicated the environment is, the more interactions the agent will need to find the optimal actions at the given state that maximize the long-term reward.

Autonomous vehicle maneuvers have been studied exhaustively using RL methods. For example, References [32,33] proposed a lane-keeping model using RL, while lane change maneuvers were performed using RL in [34], where the trained agent could perform a proper lane change under unforeseen scenarios. Reference [35] proposed a recurrent architecture for a DRL approach to execute an on-ramp merge safely. A multiple-objective RL method capable of lane keeping and performing an overtaking maneuver with collision avoidance is proposed in [36]. On the other hand, using RL to perform a platoon-related task is relatively new. Reference [37] introduced an RL scheme to find the optimal path for autonomous vehicles to form a platoon. The authors used the greedy Q-learning technique to find the optimal path that reduces energy consumption. In [38], the authors proposed a hybrid RL technique with a genetic algorithm (GA) method to control platoon formation and reduce traffic congestion and fuel consumption. GA is adopted to enhance the exploration stage of training, reduce computational costs, and accelerate the convergence rate. In [39], the longitudinal control of platoons is studied. The proposed RL approach reduced the traffic oscillations by up to 42%.

**Table 1:** State-of-the-art articles on platoon control maneuvers.

References	Vehicle Dynamics	Environment	Evaluation	Application	Control Technique
[12]	Longitudinal	MATLAB	Fuel consumption	On-ramp merging	Optimal control
[19]	Longitudinal and lateral	Not mentioned	Controller stability	Platoon Merging	Distributed MPC
[20]	Not mentioned	VISSIM [40]	String stability	Merging and splitting of platoons	PID
[22]	Longitudinal	Not mentioned	Collision avoidance	Platoon merging facing road reduction	Distributed state feedback controller
[38]	Longitudinal	PLEXE [41] and SUMO	Fuel consumption, connectivity strength, platoon stability, platoon size, and time	Platoon formulating	Hybrid DRL
[39]	Longitudinal	SUMO	Traffic oscillation and platoon stability	Platoon longitudinal control	Soft actor–critic (SAC)
[42]	Longitudinal and lateral	AUDRIC/ Dynacar	Safety	Platoon Merging	Feedforward and feedback controller
[43]	Longitudinal and lateral	MATLAB and ROS	Safety	Platoon maneuver protocols	PID, adaptive MPC, and Lyapunov controller
[44]	Longitudinal	PLEXE	String stability	Joining and leaving platoon	Consensus-based controller
[45]	Longitudinal and lateral	SUMO	Traffic flow, average speed, and delay time	Platoons at non- signalized intersection	PPO
[46]	Longitudinal	SUMO	String and controller stability	Platoons gap closing/opening	Deep deterministic policy gradient (DDPG)
[47]	Longitudinal and lateral	MATLAB	Controller robustness	Multi-vehicle merging into platoon	Nonlinear MPC
This work	Longitudinal and lateral	Python	Fuel consumption, time, jerk, maximum jerk, and speed	Platoon merging facing road reduction	Maskable PPO

Compared to the literature, the use of RL to determine the optimal merging location has not been investigated, and this paper fills this gap by utilizing DRL to find the merging strategy that optimizes several metrics, including time to merge, jerk, maximum jerk, speed, and energy consumed.

## 3. Simulation Environment

### 3.1. Vehicle Platoon

**Figure 1:**
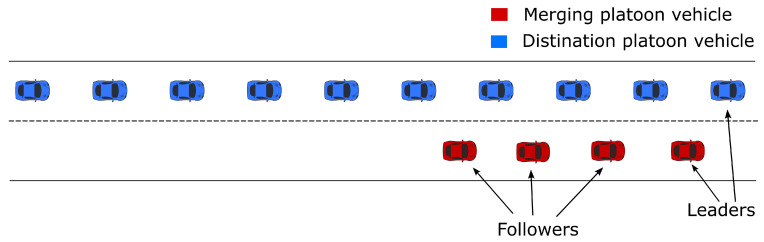
Initial platoon configuration.

The platoon configuration we consider in this paper is shown in Figure 1, where the destination platoon consists of ten vehicles to measure the impact of the merging technique (nine followers and one leader). These vehicles are initialized to be 11 m away from each other. On the other side, the merging platoon consists of four vehicles (three followers and one leader). Suppose that the lane for the merging platoon is about to end, and the goal here is to find the optimal merging location for the destination platoon. Therefore, by the end of the simulation, all the merging vehicles should be merged to the destination platoon on the other lane to form one single platoon of fourteen vehicles (thirteen followers and a single leader).

The platoon travels as one unit without the need to physically couple the vehicles of the platoon, which can be achieved by maintaining a fixed spacing distance between the platoon’s members. Two typologies are used in the literature to achieve that, i.e., *constant spacing policy* and *time headway policy*. In the constant spacing policy, the platoon ensures the desired spacing between each vehicle in the platoon regardless of the velocity of the platoon. In the headway time policy, the desired spacing changes with respect to the vehicle’s velocity so that the spacing distance is more extensive for higher velocities to ensure safety by providing more time for the follower vehicle to react to breaks. The platoon in this paper uses the constant distance spacing policy.

**Figure 2:**
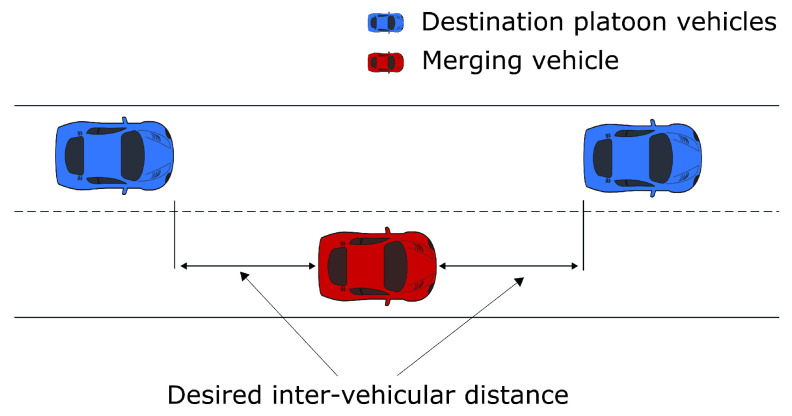
Illustration of gap generation.

At the start of the scenario, the initial speed of the vehicles and the inter-vehicular gaps are equal to their respective desired values. The desired speed, $v_{d}$, equals 10 m/s. All the merging platoon vehicles can ask to merge at any time during the simulation. Furthermore, since the platoons consist of CAVs that are capable of communicating with each other for cooperating merging when any vehicle asks to merge, a gap generation operation will be cooperatively performed by nearby vehicles in both platoons to ensure sufficient space for the merging vehicle to perform a lane change. Particularly, the controller selects the vehicles that need to increase their spacing distance so that there is a safe distance for the merging vehicle to merge into (Figure 2). The selection will be based on the position of the merging vehicle. When the gap generation operation is complete, the lateral controller of the merging vehicle will perform a lane change to merge with the destination platoon. After the merging vehicle arrives at the target lane, a platoon reformulation occurs. The reformulation reassigns the leader of the platoon to the front vehicle and the target vehicle for each vehicle.

### 3.2. Vehicle Model

**Figure 3:**
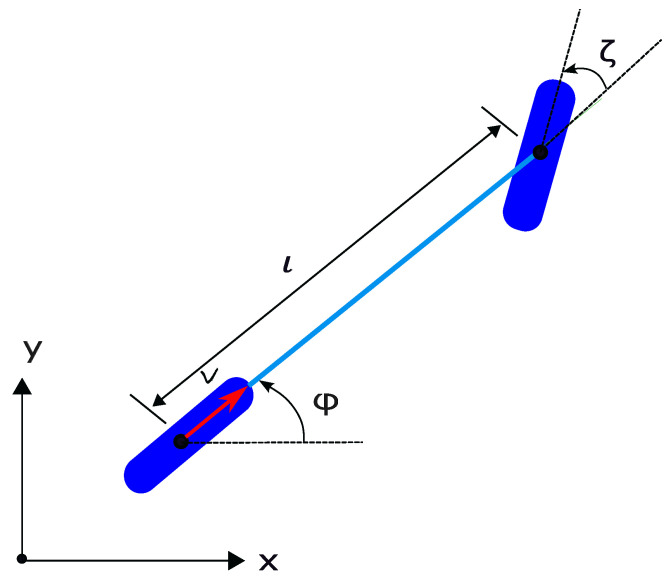
Schematics of the vehicle dynamics model.

Both the longitudinal and lateral dynamics of vehicles are taken into consideration. The vehicle dynamic model is briefly described in this section, and interested readers are referred to the relevant reference, e.g., [48,49,50]. The model of the vehicle used is depicted in Figure 3 and can be formulated as follows [48]:

(1a) $$\dot{v}=a$$

(1b) $${\dot{p}}_{x}=v\;\cos(\phi )$$

(1c) $${\dot{p}}_{y}=v\;\sin(\phi )$$

(1d) $$\dot{\phi }=\frac{v}{l}\;\tan\left(\zeta \right),$$

where $\left(p_{x},p_{y}\right)$ denotes the position of the vehicle, *l* is the wheelbase, and ϕ is the yaw angle. The control variables are the acceleration *a* and the steering angle ζ.

### 3.3. Longitudinal Control

In longitudinal control, the controller tracks the difference between the longitudinal position of the follower vehicle and the longitudinal position of its target vehicle (the vehicle in the front) to the desired value for each follower vehicle by controlling the acceleration of the vehicle. A conventional PID controller is used to control the longitudinal inter-vehicular distance between each vehicle and the vehicle in front of the same platoon. The PID is formulated as

(2) $$u_{k}\left(t\right)=k_{p}\;e_{k}\left(t\right)+k_{d}\frac{d}{dt}e_{k}\left(t\right)+k_{i}\int_{0}^{t}e_{k}\left(t\right)\;dt,$$

where $k_{p}$, $k_{d}$, and $k_{i}$ represent the proportional, derivative, and integral gain of the controller, respectively. $u_{k}\left(t\right)$ and $e_{k}\left(t\right)$ are the control variable and the error signal of the *k*th vehicle, respectively. The error signal can be calculated as

(3) $$e_{k}\left(t\right)=x_{k+1}-x_{k}-d_{ref},$$

where $x_{k+1}$ and $x_{k}$ are the longitudinal coordinates of the *k*th vehicle and its target vehicle, respectively. $d_{ref}$ represents the desired inter-vehicular distance.

### 3.4. Lateral Control

When the gap generation operation is complete, the merging vehicle generates a lane-changing path and follows it to the other lane. Using Bézier curves to generate the reference trajectory results in smoother routes that are easy to track by the merging vehicles [51]. With n+1 control points, a Bézier curve of order *n* is formulated as described by [51]

(4) $$P_{\left[t_{0},t_{t1}\right]}\left(t\right)=\sum_{i=0}^{n}B_{i}^{n}\left(t\right)\;P_{i},$$

where $P_{i}$ are control points, and $B_{i}^{n}\left(t\right)$ is the Bernstein polynomial given by

(5) Bin(t)=ni(t1−tt1−t0)n−i(t−t0t1−t0)ii∈{0,1,...,n}

**Figure 4:**
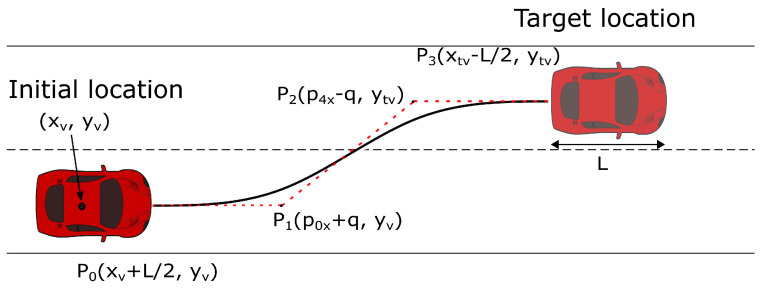
Lane changing cubic Bézier curve.

A Bézier curve has several unique properties, but the most satisfactory for lane-changing maneuvers is that the curve’s starting and ending segments are tangent to the first and last points. Therefore, the line between the first two control points and the line between the last two control points can be selected to be parallel to the lanes (Figure 4). By doing this, at the end of the lane change, the vehicle will have the same heading angle as the lanes.

A third-order Bézier curve with four control points ($p_{0}$, $p_{1}$, $p_{2}$, and $p_{3}$) is used in this paper. Therefore, (4) reduces to

(6) $$P\left(t\right)={\left(1-t\right)}^{3}P_{0}+3t{\left(1-t\right)}^{2}P_{1}+3t^{2}\left(1-t\right)P_{2}+t^{3}P_{3},$$

with t∈[0,1]. As shown in Figure 4, the first and last control points ($P_{0}$, $P_{3}$) are positioned at the front of the merging vehicle and the back of its target vehicle, respectively. The orientation of lines $P_{0}P_{1}$ and $P_{2}P_{3}$ are parallel to the lane lines to reduce the vehicle’s post-curve adjustment time. Furthermore, setting

(7) $$q=P_{0,x}-P_{1,x}=P_{2,x}-P_{3,x},$$

yields a symmetric Bézier curve around the path center, making *q* the only hyperparameter to be tuned to obtain a smooth curve.

Similar to longitudinal control, a PID controller is used to track the lateral offset between the vehicle and the Bézier trajectory. When the merging vehicle reaches the center of the target lane, the PID lateral controller is then used to track the center line so that the vehicle will be performing lane-keeping.

## 4. Preliminary Study on Reinforcement Learning

### 4.1. Deep Reinforcement Learning (DRL)

An RL algorithm can be formed as a Markov decision process (MDP) [25,28,29,30], a statistical technique that samples from a complicated distribution and estimates its characteristics. MDP is used to choose the appropriate action given a complete set of observations [52]. When the environmental dynamics are complex to determine, at least without oversimplifying, the best way to study it is through statistics. The mechanism can be understood by sampling to find a correlation between specific events and state-action pairs. MDP is a tuple of (s,a,p,r,γ) where *s* is a set of states, *a* is the set of actions the agent can take, *p* is the transition probability matrix, *r* is the immediate reward emitted by the environment upon the receipt of the actions from the agent, and γ is the discount factor. The interactions between the agent and the environment help the policy better estimate the probability distribution of the reward when selecting an action given a particular state. For example, with the agent utilizing MDP to interact with the environment for several time steps, the policy, π, may tend to increase the likelihood of selecting the actions that maximize the cumulative discounted rewards it receives. In order to converge on the optimal policy, the agent should balance exploring and maximizing the total rewards, which is called the exploration and exploitation dilemma. The agent should start by collecting information about the environment (exploring) to make good future decisions (exploiting).

DRL combines deep neural networks and a reinforcement learning technique to help the agent increase the cumulative rewards. The need for more efficient function approximators becomes more critical with the increase in states or actions dimension. As the name indicates, deep reinforcement learning uses a deep neural network to estimate the value function of each state, which is the case in value-based reinforcement learning approaches [53]. Alternatively, the deep neural network can be used to learn the optimal policy that maps states to actions, such as the REINFORCE method [54]. Some other reinforcement learning algorithms use multiple neural networks to perform different tasks, such as the actor-critic methods [55].

### 4.2. Proximal Policy Optimization Algorithm

In this work, the proximal policy optimization (PPO) algorithm is used [14,15], which is a policy based on the policy gradient RL algorithm. In general, policy gradient methods attempt to optimize the policy directly [56]. The policy, π, is a function approximator, usually a neural network, parameterized with respect to a set of parameters θ. Essentially, gradient ascent is used to change θ towards the increase in the cumulative rewards. Policy gradient methods are significantly faster in practice [25], but they suffer from some fundamental problems. For example, the agent’s training data are based on the current policy when the data were collected, which causes the rewards and observations distribution to constantly change based on the current policy. This change leads to instability in the whole training process. Furthermore, policy gradient methods are susceptible to hyper-parameters such as entropy coefficient, learning rate, and weight initialization, to name a few. To address these issues, PPO has been proposed in the literature as a scalable, robust, and sample-efficient policy gradient algorithm that is also relatively easy to code.

For policy gradient methods, the loss is defined as follows:

(8) $$L^{PG}\left(\theta \right)=\hat{E}\left[\log\pi_{\theta }\left(a_{t}|s_{t}\right)\hat{A_{t}}\right],$$

where $\hat{E}$ is the expected return over a batch of data, $\hat{A_{t}}$ is the estimation of the advantage function at time step t, and $\pi_{\theta }$ is a stochastic policy. $\pi_{\theta }\left(a_{t}|s_{t}\right)$ is the likelihood of choosing the action *a* given the state *s*. The advantage function can be calculated as

(9) $$\hat{A_{t}}=G_{t}-V_{t}\left(s\right),$$

where $G_{t}$ is the total discounted rewards, and $v_{t}$ is a function or value estimation of the state *s*. Making multiple optimization steps on this loss using the same data collected from the environment is not advised because that might change the policy too much towards that specific trajectory. TRPO [56] has already tried to solve this issue, but their solution (trust region optimization method) includes a second-order derivative and its inverse, which is very computationally expensive. PPO solved the same problem by introducing a soft constraint that makes the objective function solvable using a first-order optimizer. The new objective function will prevent the policy from changing too much by clipping the objective value, making it possible to run multiple optimization steps on the cost function without moving the policy too far in the parameter space. The loss function proposed by PPO is as follows [15]:

(10) $$L^{CLIP}\left(\theta \right)=\hat{E}\;\left[min\left(r_{t}\left(\theta \right)\;\hat{A},clip\left(r_{t}\left(\theta \right),1-\epsilon ,1+\epsilon \right)\right)\hat{A}\right],$$

where $r_{t}$ is the probability ratio of the policy before the new policy and the policy before the update $\pi_{\theta_{old}}\left(a_{t}|s_{t}\right)$. The epsilon is a hyperparameter that defines how much an update can change the policy. In the PPO algorithm, the agent collects data by interacting with the environment. Next, the advantage estimate of each state is calculated. Finally, for *k* epochs, the stochastic gradient descent is applied with *N* mini-batches of the collected data to update the policy. A pseudocode of the PPO algorithm is shown in Algorithm 1.

**Figure 5:**
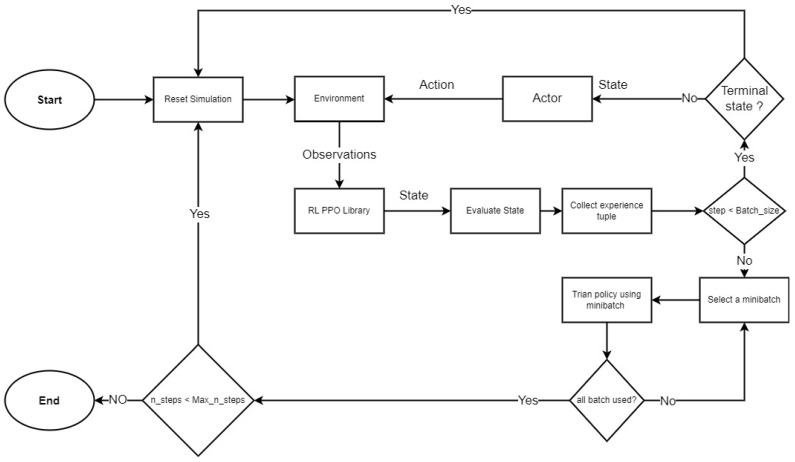
Data flow diagram of the PPO algorithm.

Finally, Figure 5 shows a flow chart of the PPO algorithm.
**Algorithm 1:** PPO, Actor–Critic Style [15] **for**
iteration 1,2, ...
**do**  **for**
actor=1,2, ... N
**do**   Interact with the environment using the $\pi_{\theta_{old}}$ policy   *feed the states to the critic network to calculate states*    base estimate   Compute advantage estimates  **end for**  Optimize surrogate *L* wrt θ, with *K* epochs  update the policy **end for**

## 5. RL-Based Merging Strategy

### 5.1. States Observation and Action Space

**Figure 6:**
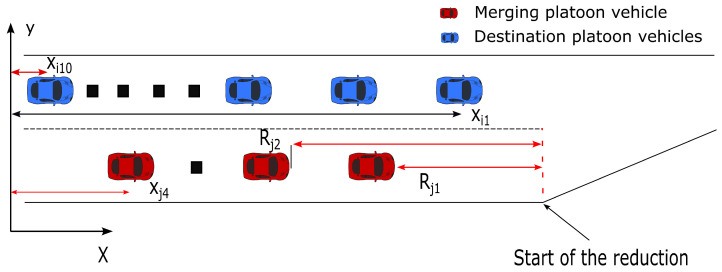
Observation space measurements.

In this simulation, there are fourteen autonomous vehicles. Four are in the merging platoon, and the rest belong to the destination platoon. The states should describe all the essential information about every vehicle so that the agent can have enough information to take reasonable actions. The global *x* position of every vehicle is provided, and the relative distance of each merging vehicle to the start of the road reduction is provided, as shown in Figure 6. The state of whether every merging vehicle is merged or not is also fed to the network. It can be observed that there are continuous and discrete attributes, and each has its own maximum and minimum values, meaning that in order to achieve a fast convergence, normalization is inevitable. The state vector can be formed as follows:

(11) $$S=\left[\begin{array}{c} p_{x_{i1}}\\ \vdots \\ p_{x_{i10}}\\ p_{x_{j1}}\\ \vdots \\ p_{x_{j4}}\\ R_{j1}\\ \vdots \\ R_{j4}\\ s_{j1}\\ \vdots \\ s_{j4} \end{array}\right],$$

where *i* and *j* denote the destination and merging platoon vehicles, respectively, as shown in Figure 6. $p_{x}$ is the global *x* coordinate, *R* is the relative distance between the corresponding vehicle and the starting point of the road reduction, and *s* is the status of the vehicle as follows:

(12) $$s_{v}=\left\{\begin{array}{cc} 1, & =>\mathrm{if}\;\mathrm{vehicle}\;v\;\mathrm{is}\;\mathrm{merged}\\ 0, & =>\mathrm{if}\;\mathrm{vehicle}\;v\;\mathrm{is}\;\mathrm{not}\;\mathrm{merged} \end{array}\right.$$

Since we have four vehicles in the merging lane, the action space size is four, one for each vehicle. The action should stimulate the corresponding merging platoon vehicle to ask to merge with the other lane. There are two options for the action space: discrete or continuous. The continuous option means that the agent will select a relative distance at the start of the simulation to ask to merge and ultimately try to find the optimal distance. That approach works only if perfect prediction of the future behavior of all vehicles is available. On the other hand, with the discrete action space, the agent will make real-time actions based on the observations it is receiving. The action for the vehicle *v* is as follows:

(13) $$a_{v}=\left\{\begin{array}{cc} 1, & =>\mathrm{Request}\;\mathrm{to}\;\mathrm{merge}\\ 0, & =>\mathrm{Stay}\;\mathrm{in}\;\mathrm{the}\;\mathrm{same}\;\mathrm{lane} \end{array}\right.$$

### 5.2. Rewards Functions

In this paper, different reward functions will be used to train the RL model to investigate their impact on the merging strategy. The vehicles’ time consumed, energy consumption, mean jerk, maximum jerk, and relative position are characteristics used to incentivize or discourage the agents’ decisions. The first important index is that all the vehicles merge with the not-ending lane and do not crash. A penalty of negative rewards is returned to the RL algorithm for every non-merged vehicle that gets close to the start of the road reduction.

#### 5.2.1. The Energy Consumption

**Figure 7:**
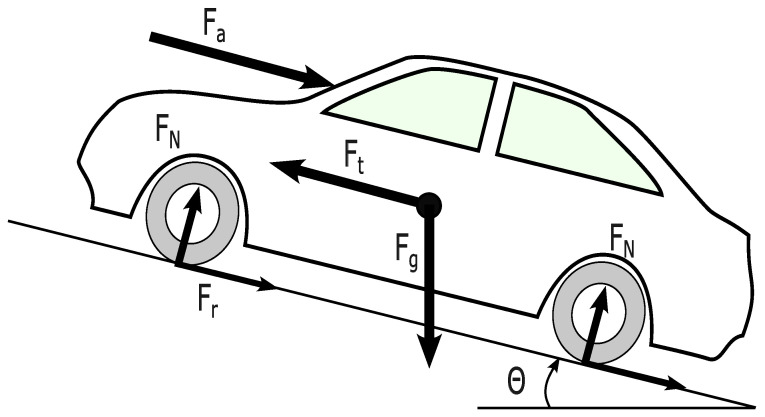
Free body diagram of the vehicle.

The amount of energy consumed during the maneuver is essential in evaluating the model behavior. In this work, an electric vehicle energy model is used to calculate the energy consumed by all vehicles to finish the merge. Using Newton’s second law, the forces on the wheel can be formed as follows in Equation (14).

(14) $$\sum F_{x}=m\;a,$$

where *a* is the vehicle acceleration, *m* is the mass of the vehicle, and $F_{x}$ is the summation of forces applied on the vehicle in the *x* direction. Substituting the forces shown in Figure 7 is expressed in Equation (15).

(15) $$F_{t}-F_{a}+F_{g}+F_{r}=m\;a,$$

where $F_{a}=0.5\;C_{d}\left(D\right)\;\rho \;A\;v^{2}$ is the aerodynamic resistance, $F_{r}=m\;C_{r}\;g\;\cos\left(\theta \left(t\right)\right)$ is the friction force, $F_{g}=m\;g\;\sin\left(\theta \left(t\right)\right)$ is the gravity force, and $F_{t}=m\;a_{w}$ is the traction force. Note that here $a_{w}$ is the wheel acceleration, *A* is the frontal area of the vehicle, ρ is the air density, *g* is the acceleration of gravity, θ(t) is the gradient of the road, $C_{d}$ is the air drag coefficient, $C_{r}$ is the rolling resistance coefficient, *g* is the gravity acceleration, *a* is vehicle acceleration, and *D* is the relative distance between the vehicle and the vehicle in front of it [57].

Reorganizing and substituting the force formulas in Equation (15) yields:

(16) $$a_{w}=a+\frac{0.5\;C_{d}\left(D\right)\;\rho A\;v^{2}}{m}+C_{r}\;g\;\cos\left(\theta \left(t\right)\right)+g\;\sin\left(\theta \left(t\right)\right)$$

This work adopts a 2019 Nissan LeafSV EV from [12]. The energy consumption of the vehicle during the simulation time ts is as follows:

(17) $$R_{e}=\int_{0}^{ts}\left(m\;a_{w}\;v+\frac{b\;{\left(mr_{t}\right)}^{2}}{\xi^{2}}\;a_{w}^{2}\right)\;dt,$$

where ξ is the gear ratio, $r_{t}$ is the radius of the tire, and *b* is the motor loss coefficient, measured experimentally.

#### 5.2.2. The Vehicle Jerk

Passenger comfort has been studied thoroughly, especially for automated vehicles, as it can affect the adoption of autonomous vehicles. Repetitive exposure to low-frequency motions can develop motion sickness [58], and regular exposure to high-frequency motions can lead to lower back pain [59,60]. The jerk can be used to sense these discomforts and sudden acceleration changes and ultimately optimize the autonomous vehicle’s behavior to ensure comfortable driving. This work uses the mean and the maximum jerk as reward functions to train the RL agent. For the mean jerk, the absolute value of the jerk of every vehicle is calculated, and the mean is sent as the reward. The reward function is expressed as follows:

(18) $$R_{j}=\frac{-1}{N}\sum_{n=1}^{N}\left(\frac{a_{n,k}-a_{n,k-1}}{dt}\right),$$

where $R_{j}$ is the step rewards, *k* is the time step, and *N* is the number of vehicles.

For the maximum jerk as a reward function, only the maximum jerk of all vehicles is returned as the step reward. In this case, the reward function is expressed as:

(19) $$R_{mj}=-\left|\right|J_{k}{\left|\right|}_{\infty },$$

where $J_{k}$ is a vector of the absolute values of all the vehicles’ jerk at time step *k*.

#### 5.2.3. Time

Another metric used to train the RL is time. Reducing the time it takes all the vehicles to finish the merge reduces traffic congestion. For every time step, a negative reward will be sent to the RL agent until all of the merging platoon vehicles have already merged to the other lane. This will incentivize the agent to merge all the vehicles as soon as possible.

(20) $$R_{t}=\left\{\begin{array}{cc} -r, & =>\mathrm{if}\;\mathrm{merging}\;\mathrm{vehicles}\;\mathrm{did}\;\mathrm{not}\;\mathrm{merge}\;\mathrm{yet}\\ 0, & =>\mathrm{if}\;\mathrm{all}\;\mathrm{merging}\;\mathrm{vehicles}\;\mathrm{have}\;\mathrm{merged} \end{array}\right.$$

#### 5.2.4. Speed

Another reward function is proposed to encourage the model to get all the vehicles to go through the merge faster. A relative position (longitudinal velocity) of the last vehicle in the destination platoon is returned to the agent at each time step. The reward function can be obtained as follows:

(21) $$R_{s}=x_{v_{l},k}-x_{v_{l},k-1},$$

where $x_{v_{l},k}$ is the global *x* position of last vehicle in the destination platoon at time step *k*.

### 5.3. Maskable PPO

Based on the nature of our simulation, the valid actions change based on the state of the environment. Therefore, for example, a gap generation operation will start when one of the vehicles asks to merge. The vehicle’s state will be changed accordingly to “merged”, which means the agent should not be able to ask a vehicle to merge again after it is already merged into the target lane. That means for the rest of the simulation, the only proper action for a merged vehicle is “stay in the same lane”.

There are three methods to solve this problem.

- The first one is to build the simulation environment to ignore invalid actions. However, this method is not sampling-efficient since sampling ignored actions that do not affect the environment will waste a significant amount of time.
- In the second approach, a negative reward is set to penalize choosing an invalid action so that the agent will eventually learn only to select valid actions. This method will add an unnecessary complication for the policy to learn, increasing the required convergence time.
- In the third approach, a mask is used to block invalid actions, allowing the policy to only choose within the available valid actions at that state. In [61], the theoretical justification for using masking in policy gradient methods is proved.

**Figure 8:**
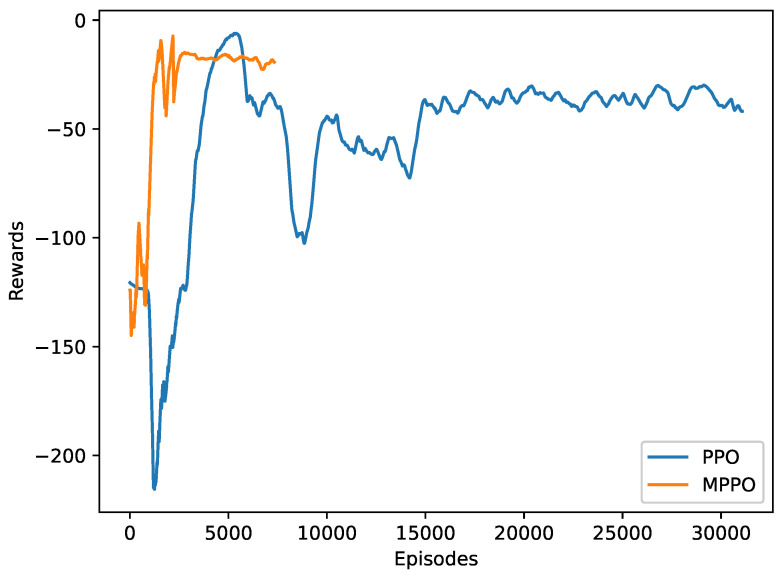
Comparison of training progress of MPPO and PPO.

All three approaches have been implemented in this work, and it was determined that the third approach, namely, Maskable PPO, performs the best. Figure 8 shows the difference in convergence time between a regular PPO, where the environment ignores invalid actions, and maskable PPO (MPPO). All numerical results presented in the rest of this paper are collected using MPPO.

## 6. Numerical Results and Discussion

A series of tests were performed to evaluate the performance of the proposed framework using our recently developed object-oriented toolbox for Python. The system includes a collection of tools and interfaces for simulating and displaying the movement of the vehicles within an intelligent transportation system environment. It also calculates the performance indices to evaluate the merging technique of the trained model. The toolbox consists of two main components, the DRL model and the environment. The PPO algorithm represents the DRL model. However, there are multiple implementations for the PPO algorithm (DRL model) [62]. Therefore, the maskable PPO from [14] is adopted in this work. The environment is created to be an OpenAI Gym class [63], with built-in functions to perform the low-level controllers to manage the simulation of non-RL-related actions. The toolbox operation consists of two stages, training and evaluation. After training, the model will be used to predict actions, and a test scenario will be simulated to evaluate the performance indices of the model behavior. This section presents numerical results. We start with single objective RL, followed by the multi-objective RL that combines all important metrics. Table 2 lists all the parameters used in this simulation. Simulations were performed with high-performance computing (HPC) nodes running Red Hat Enterprise Linux release 8.6 (Ootpa) with 192 GB of RAM and 40 CPU Cores at 2.50 GHz. The training time of a maskable PPO model with 2048 steps for each rollout and 64 batch size is 6000 episodes, around 10 h. On the other hand, the time required to simulate an entire scenario and evaluate the model actions is 400 ms.

### 6.1. Single Objective RL

Evaluation results of each single objective RL model are summarized in Table 3, where the “Simple Early Merge” model is a manually designed merging strategy for benchmarking. Specifically, this simple merging behavior would make all vehicles ask to merge at the start of the simulation, yielding a zipper-like configuration of the resultant single platoon. Detailed discussions on the results are given as follows.

#### 6.1.1. Results for Minimizing Energy Consumption Only

**Figure 9:**
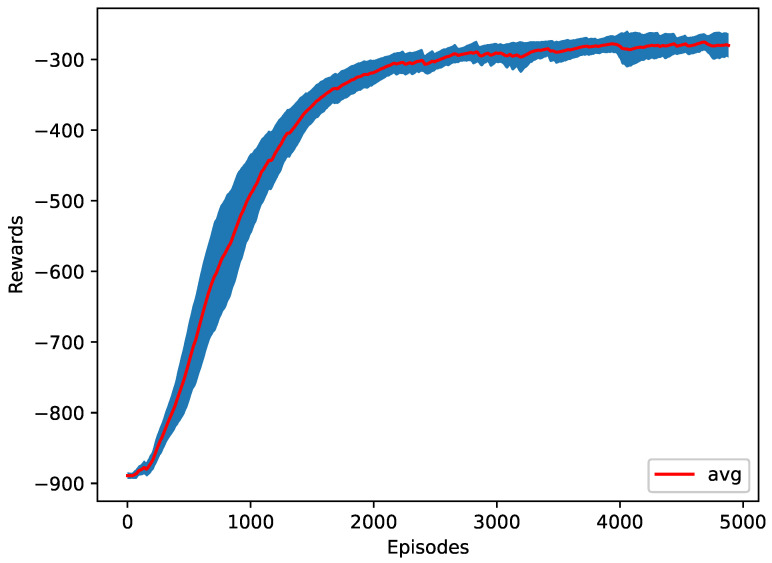
The training plot of the energy as a reward function.

For the first case, the RL agent is trying to reduce the energy consumed by the vehicles. The average of all the vehicles’ energy consumed is returned every time step. As shown in Table 3, the RL agent reduced the energy to around 21.24 MJ, which is more than 76% better than doing an early merge of all the vehicles at the start of the simulation. Furthermore, the average jerk is also significantly reduced. The RL model performed a zipper merge, starting with the first vehicle of the merging platoon asking to merge and the last vehicle of the merging platoon being the last one to ask to merge. Figure 9 shows the training progress of ten different seeds.

#### 6.1.2. Results for Minimizing the Time Required to Finish the Merge

**Figure 10:**
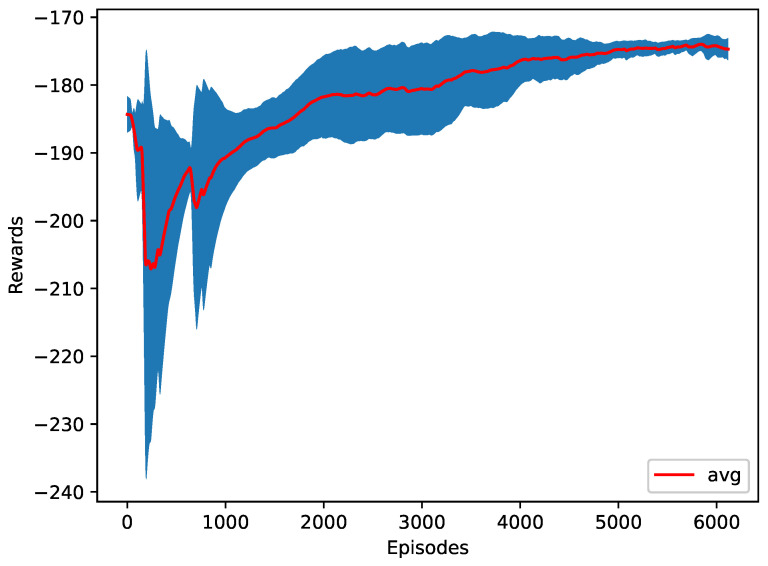
The training plot of the time as a reward function.

The RL agent is trying to reduce the time required to merge all the vehicles into one platoon. The apparent attempt to minimize the time required to finish the merge is to start merging as soon as possible to minimize the time required. However, the RL agent found a better cooperative behavior that does not merge all vehicles at the start. Instead, some vehicles surprisingly wait some time before asking to merge, which proves that the pattern or behavior of merging significantly affects the merging performance. As a result, the agent learns to perform an early zipper merge to finish in only 17.4 s, as shown in Table 3. The training progress is shown in Figure 10. It can be observed that even with the untrained model (random actions), the time rewards achieved are relatively good. The reason is that at each time step, the agent has two options for each vehicle, merge or stay in the same lane, which makes the starting probability of each action to be chosen by the agent 50%. Given that the agent will be asked to choose an action ten times every second, it is very likely that the agent will ask all the vehicles to merge in the first second.

#### 6.1.3. Results for Maximizing the Speed of the Last Vehicle in the Destination Platoon

**Figure 11:**
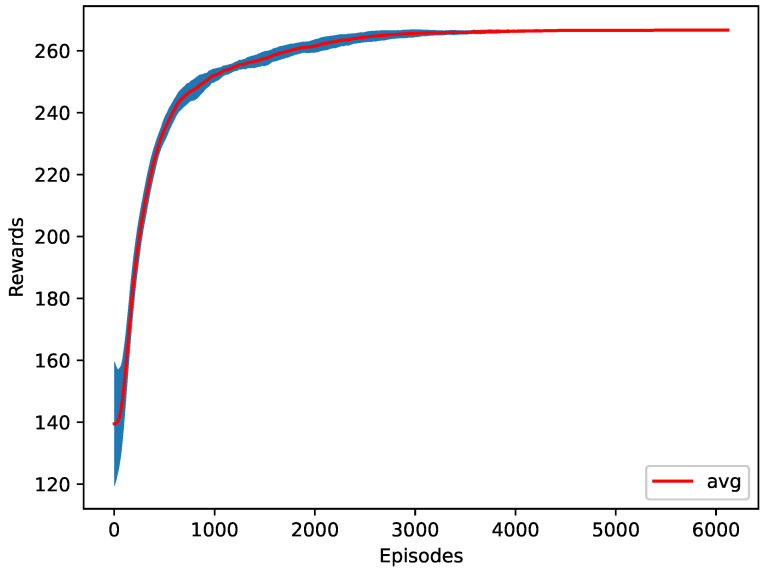
The training plot of the speed as a reward function.

In the third case, the change in the *x* position of the destination platoon’s last vehicle is returned to the agent. The change in the *x* position represents the longitudinal velocity. Increasing the longitudinal speed of the last vehicle increases the traffic flow. The agent’s average speed of the last vehicle is 8.8 m/s, where the desired speed of the last vehicle is set to 10 m/s. Figure 11 shows the training progress of ten different seeds. It is worth noting that, in this case, the last vehicle’s speed is actual lower that the case of $R_{t}$. This is likely due to the fact that rewarding based on one single vehicle can take a longer time for RL to converge and there can be multiple local optima for the RL training algorithm. However, as can be seen from Table 3, $R_{s}$ did perform better than the cases of the benchmark Simple Early Merge model and $R_{mj}$.

#### 6.1.4. Results for Minimizing the Mean Jerk of All the Vehicles

**Figure 12:**
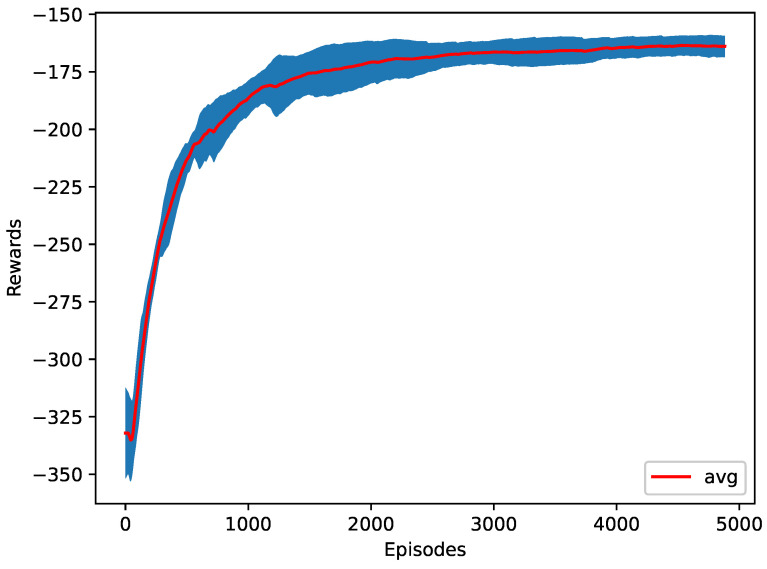
The training plot of the average jerk as a reward function.

In the fourth case, the RL agent reduces the changes in acceleration and/or deceleration of the vehicles. The average jerk of all of the vehicles is returned to the agent. The RL learned to merge in 28.8 s with only 0.4478 m/s${}^{3}$ average jerk. Reducing the average jerk reduces the vehicle’s changes of acceleration and deceleration and, therefore, decreases energy consumption by more than 51% less than a regular early merge. Figure 12 shows the training progress of ten different seeds.

#### 6.1.5. Results for Minimizing the Maximum Jerk

**Figure 13:**
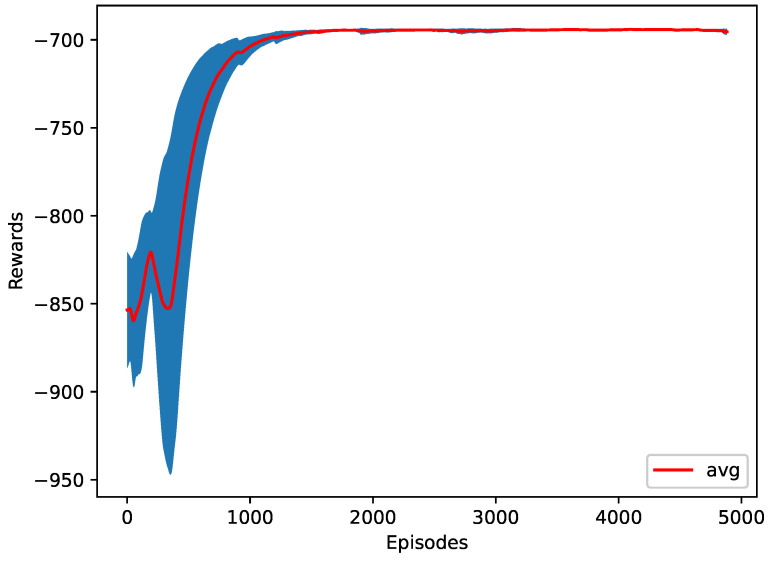
The training plot of the maximum jerk as a reward function.

Reducing the average jerk does not necessarily mean that the jerk is satisfied for every vehicle at each time step. In the fifth case, the RL is encouraged to reduce the maximum jerk of all vehicles. The maximum value of all the vehicles’ jerks is returned to the agent at every time step. The RL agent successfully reduced the maximum jerk to 2.3019 m/s${}^{3}$, 5.5%, and decreased the time spent by 36.8%, which is better than the average-jerk RL. This comes with the cost of increasing energy consumption by 51% and the average jerk by 52.7%. The training progress of ten different seeds is shown in Figure 13.

### 6.2. Multi-Objective RL

**Figure 14:**
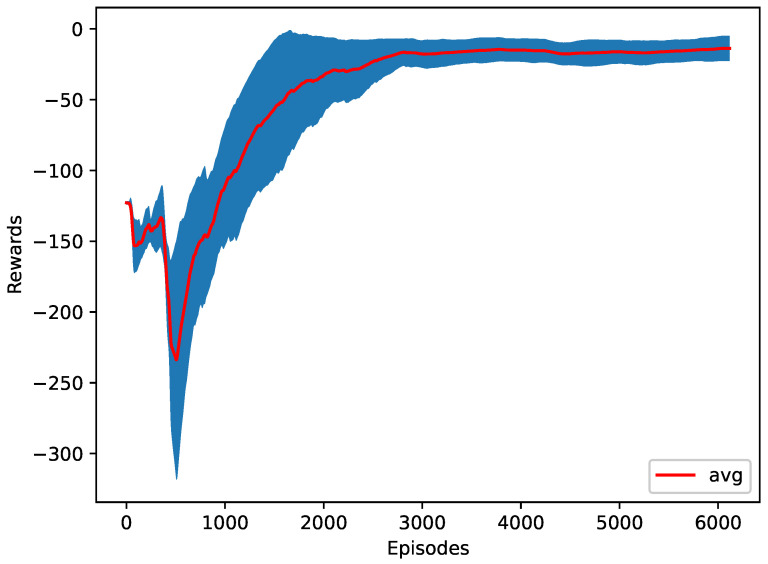
The training plot of the multi-objective reward function (Case 1).

**Figure 15:**
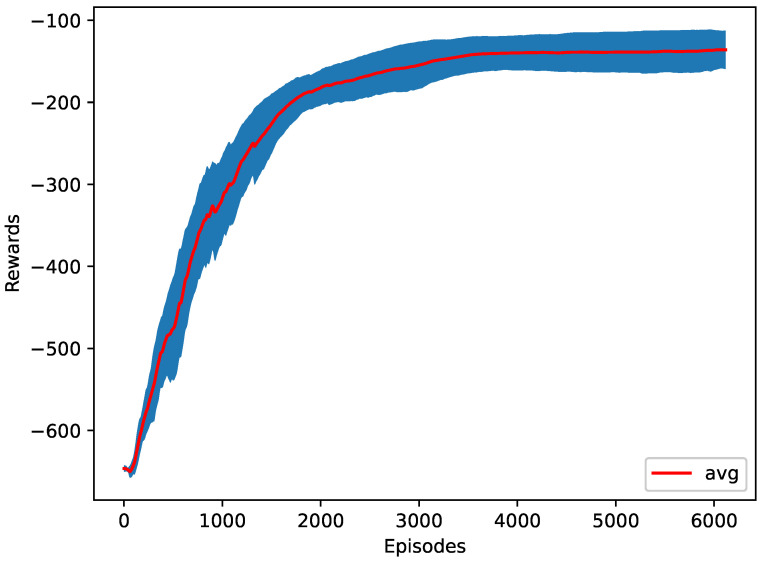
The training plot of the multi-objective reward function (Case 2).

It can be observed that when the average jerk is minimized, the agent takes too long to finish the merge, but when time is the main objective of the RL, the energy consumed and the jerk increase. This means there should be a balance based on the type of drive required. A weighted sum of all the individual rewards is returned to the agent every time step, which can be formulated as follows:

(22) $$R_{\mathrm{multi}-\mathrm{objective}}=-\delta_{1}R_{t}+\delta_{2}R_{s}-\delta_{3}R_{j}-\delta_{4}R_{e},$$

where $\delta_{1,2,3,4}\in \left[0,1\right]$ are the weights. $R_{e}$, $R_{j}$, $R_{t}$, and $R_{s}$ are formulated in Equations (17), (18), (20), (21), respectively. Figure 14 and Figure 15 show the training progress of ten different seeds with weights $\delta_{1}=0.2,\delta_{2}=0.1$, $\delta_{3}=0.3,\delta_{4}=0.3$ (Case 1) and $\delta_{1}=0.1,\delta_{2}=0.1,$$\delta_{3}=0.3,\delta_{4}=1$ (Case 2), respectively. The agent will accommodate with regard to time and energy for the first set of weights while maintaining a low amount of mean jerk, while the agent for Case 2 will care more about energy, which increases the time by a few seconds, as shown in Table 4, which lists an additional result for Case 3 with $\delta_{1}=0.2,\delta_{2}=0.1,\delta_{3}=0.4,\delta_{4}=0.4$. As can be seen from Table 4, the merging strategy is greatly influenced by the weights for (22). Usually, the balance of each metric is up to the policymaker, and the proposed framework is flexible to accommodate a variety of merging strategies by simply changing the weights of (22) and, hence, avoiding manual control design for each scenario.

## 7. Conclusions

This paper shows that deep reinforcement learning (DRL) is a promising approach to control different aspects of the behavior of connected and automated vehicles (CAV) approaching a lane reduction. This work investigates the cooperative merging of two platoons of electrified CAV during a lane reduction. A DRL framework is proposed to learn the optimal merging policy to best utilize road capacity while ensuring safety and passenger comfort. To minimize the time, energy, and average jerk required for connected vehicles, actor–critic style maskable proximal policy optimization is used to predict the distance at which the merging vehicle should request to merge, and we employed a Bézier curve and a PID controller to handle low-level control tasks and produce an optimized driving behavior. Using PPO-based deep reinforcement learning, we were able to train a model that can find the appropriate actions based on the current driving conditions and the surrounding vehicles, resulting in a reduction of 76.7% in energy consumption and 50% in average jerk. The results show that the time at which vehicles merge can significantly affect the traffic flow, energy consumed, and the passengers’ comfort.

For future work, due to modern highways often having three lanes, this method could be used as a foundation for exploring 3–2 or 3–1 road reduction scenarios. Furthermore, a cooperative multi-agent reinforcement learning algorithm could be used instead of a single centralized controller, which is vulnerable to communication failures or latency. A transportation simulator, e.g., SUMO [64], will be used to investigate the effect of the proposed RL-based cooperative merging strategy on large-scale traffic. In addition, it is important to note that platoon merging requires cooperation from all vehicles in order to function correctly. However, this may not always be feasible in real traffic situations, as some vehicles may choose not to cooperate or may not have the ability to communicate. Therefore, a more complicated scenario should be considered.

## Figures and Tables

**Table 2:** Simulation parameters.

Parameters	Value	Description
N	14	Total number of vehicles
*r*	1	Time model negative reward
q (m)	6	Distance between control points to obtain a smooth Bézier curve
$\theta_{a}$	22 × 64 × 64 × 8	Actor Network Architecture
$\theta_{c}$	22 × 64 × 64 × 1	Critic Network Architecture
$v_{d}$ (m/s)	10	Leaders desired speed
$d_{ref}$ (m)	11	Desired inter-vehicular distance
Batch Size	64	Number of tuples propagate the network
ϵ	0.2	Clipping hyperparameter
Number of Epochs	10	How many times experiences are used to train the network
RL discount factor γ	0.99	Defines the priority of immediate rewards
*l* (m)	4	Wheelbase length
*m* (kg)	1618.87	Mass of the vehicle
ρ (kg/m${}^{3}$)	1.28	Air density
*A* (m${}^{2}$)	2.5334	Frontal area of the vehicle
θ(t) (rad)	0	Gradient of the road
*g* (m/s${}^{2}$)	9.81	Acceleration of gravity
$C_{r}$	0.015	Rolling resistance coefficient
ξ	8.193	Gear ratio
*r* (m)	0.4318	Radius of the tire
*b*	1.0355	Motor loss coefficient
$k_{p}$	0.2	Proportional gain
$k_{d}$	0.7	Derivative gain
$k_{i}$	0.00034	Integral gain

**Table 3:** Evaluation results of different single objective RL models.

RL Model	$R_{e}$	$R_{j}$	$R_{\mathrm{mj}}$	$R_{s}$	$R_{t}$	Simple Early Merge
Energy Consumed (MJ)	**21.24**	44.76	91.5	92.77	80.2	91.5
Average Jerk (m/s${}^{3}$)	0.4907	**0.4478**	0.6841	0.692	0.634	0.6841
Maximum Jerk (m/s${}^{3}$)	2.95	2.436	**2.3019**	3.09	2.565	**2.3019**
Last Vehicle’s Average Speed (m/s)	7.67	6.17	8.869	8.865	**8.91**	8.869
Time (s)	27.1	28.8	18.2	18.1	**17.4**	18.2

**Table 4:** Evaluation results of the multi-objective RL with different weights.

	Case 1	Case 2	Case 3
Max Jerk (m/s${}^{3}$)	3.01	**2.6**	3.35
Avg Jerk (m/s${}^{3}$)	**0.52**	0.54	0.57
Last Vehicle’s Average Speed (m/s)	7.75	**8.9**	7.97
Energy Consumed (MJ)	56.66	**46.48**	83.68
Time (s)	25.5	**21.6**	22.4

## Data Availability

The data presented in this study are available on request from the corresponding author. The data are not publicly available due to restriction.
